# Development of Biomimetic Gelatin-Hydroxyapatite Composites Containing Doxycycline with Osteogenic Potential

**DOI:** 10.1021/acsabm.5c00355

**Published:** 2025-07-16

**Authors:** Sandra Daniela Motta, Pedro Henrique Passos Leite, Alexia David Nascimento, Rubén Dario Sinisterra, Maria Esperanza Cortés

**Affiliations:** 1 Technological Innovation, ICEX, 28114 Universidade Federal de Minas Gerais (UFMG), Av. Antônio Carlos, 6627, Belo Horizonte, Minas Gerais, CEP 31270-901Pampulha, Brazil; 2 Chemical Department, ICEX, 28114 Universidade Federal de Minas Gerais (UFMG), Av. Antônio Carlos, 6627, Belo Horizonte, Minas Gerais, CEP 31270-901 Pampulha, Brazil; 3 Restorative Dentistry Department, Dentistry Faculty, 28114 Universidade Federal de Minas Gerais (UFMG), Av. Antônio Carlos, 6627, Belo Horizonte, Minas Gerais, CEP 31270-901 Pampulha, Brazil

**Keywords:** gelatin, hydroxyapatite, doxycycline, composite, scaffold, drug release, osteogenesis

## Abstract

Worldwide, bone illnesses and disorders are on the rise. Artificial bone substitutes may replace autogenous bone transplants. Due to its cytocompatibility, nontoxic breakdown, infinite supply, and disease resistance, biopolymers are becoming more attractive in biomedical applications. Most graft materials used in composites lack the antibacterial properties necessary for matrix biocompatibility. This study aims to develop two hybrid polymeric composites incorporating doxycycline to enhance cell compatibility and antibacterial activity, using gelatin–Hydroxyapatite-gelatin (HA-gel) cross-linked with 1% w/v (1-ethyl-3-(3-(dimethylamino)­propyl)) carbodiimide (EDC) and 0.3, 0.7, and 1.2% doxycycline. The gelatin–hydroxyapatite scaffold with 1% EDC cross-linker and 0.3% doxycycline (1.1) demonstrated reduced cytotoxicity in L929 fibroblasts and MC3T3 preosteoblastic cells. At 21 days, scaffolds increased preosteoblastic cell (MC3T3) proliferation, cell adhesion, and ALP. The *in vitro* drug release profiles and steady enzymatic biodegradation are consistent with the bone regeneration time. Doxycycline-enhanced gelatin-HA composites have porosity, controlled breakdown, and swelling, making them biocompatible for bone tissue regeneration. Doxycycline increased preosteoblastic cell proliferation, offered antibacterial characteristics, and regulated breakdown to meet bone repair timelines. The composite improved cell adherence and regulated drug release, making it a viable tissue engineering and drug delivery medium.

## Graphical abstract

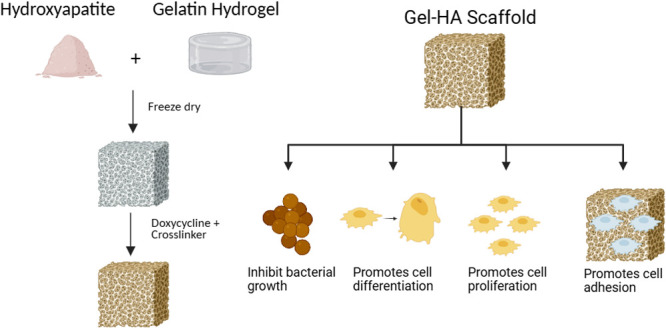

## 1 Introduction

The global incidence of bone diseases and disorders has shown a sharp upward trend, especially in populations where aging is accompanied by increased obesity and low physical activity.^1^ With an estimated 2,000,000 bone graft procedures performed each year, bone repair structures continue to be a promising line of research due to the increasing demand and low stock of bone substitutes.^2^

Bone has an intrinsic repair capacity when traumatic, pathological, and surgical defects are smaller than what has been defined as critical size defects (<5 cm). Larger failures can still be healed with the support of biodegradable biomaterials with osteoconductive or osteogenic properties.^3^ Artificially developed bone tissue has been seen as a potential alternative to the conventional use of bone grafts, due to its unlimited supply and nontransmission of diseases.^1^ Human tissues are mainly composed of cells and an extracellular matrix (ECM), which plays a fundamental role in providing a strong and flexible network, activating and stimulating specific cellular functions, and promoting greater growth.^4^

Recently, the field of tissue engineering (TE) has increased exponentially due to the growing demand for artificial tissues.^5^ Biopolymers, or polymers made from natural resources, are becoming increasingly popular for biomedical applications due to their many advantages, including cytocompatibility and nontoxic degradation.^6^

Currently, in the field of tissue engineering (TE), researchers are exploring various extracellular matrices (ECM), such as films, nanoparticles, hydrogels, and three-dimensional (3D) structures composed of biopolymers, for both in vitro and in vivo applications.^7–9^ However, artificial ECMs must be both mechanically and biologically functional, optimizing the material to perform specific functions and achieve similarity to human tissue^10^

Biopolymeric composites are artificial structures, organic or synthetic, with predetermined physicochemical characteristics and porosity. Most polymeric materials that contribute to the development of composites generally do not provide the desired mechanical and degradation properties for the matrix, because they have few links between the polymeric chains. Therefore, cross-linking is necessary to create covalent bonds between the polymeric chains creating three-dimensional polymers with high molecular weight. Cross-linking is a simple method in which chemical or physical bonds are established between polymeric chains to modify the mechanical, biological, and degradation properties of polymeric materials.^6^ Among all cross-linking methods, chemical cross-linking seems to be the most common and effective. With EDC being one of the most commonly used cross-linkers for collagen, it improves its mechanical and biodegradability properties by linking amino and carboxyl groups, with several studies supporting these characteristics.^11–14^ In addition, when designing composite materials, several important factors must be considered. These include the selection of an appropriate biomaterial, fine-tuning its microstructure, assessing its biological efficacy, and understanding the physicochemical properties of the cross-linking reagents.^6^ As an example, gelatin (gel) is made when native collagen is partially hydrolyzed. It is nontoxic, noncarcinogenic, biocompatible, and biodegradable. It finds wide applications in the pharmaceutical and medical fields, such as materials for dressings, tissue engineering composites, and drug delivery vehicles.

Gelatin, a product of partial hydrolysis of native collagen and characterized by its nontoxicity, noncarcinogenicity, biocompatibility, biodegradability, and promotes a good environment for cell growth and adhesion,^15^ can serve as the basis for composite preparations. Pharmaceutical and medical fields widely use this, incorporating it into materials for dressings, tissue engineering composites, and drug delivery vehicles. However, composites prepared from gelatin show weak mechanical and hydrolysis resistance, along with an immediate release of drugs associated with them.

Gelatin composites possess a 3D porous structure that benefits cell adhesion.^7^ However, they exhibit weak mechanical resistance and are susceptible to hydrolysis. Cross-linking stabilizes gelatin composites, enhancing their stability during implantation.

Hydroxyapatite (Ca_10_(PO_4_)­6­(OH)_2_, HA) is a natural mineral that constitutes a large amount of inorganic compounds in the bone matrix. Designing a gelatin composite with hydroxyapatite can enhance composite stability. Gelatin can coat porous HA as an example of a simple biphasic composite, enhancing the material’s mechanical properties and improving drug release.

One of the ways found to improve these characteristics is the synthesis of hydroxyapatite in the gelatin matrix, which improves the physicochemical properties of the polymeric network.^15^ In addition, the local administration of antimicrobial drugs significantly improves the healing and regeneration of new tissues.^16^

Loading drugs within composites offers significant advantages, as the material-drug system with local administration significantly improves tissue healing and regeneration. Therefore, the goal of this research is to develop hydroxyapatite-gelatin (HA-gel) composites that contain doxycycline, which can be obtained through a biomimetic approach and has the potential to promote osteogenic growth. Hydrogels mimic the extracellular matrix and promote tissue growth more favorably than polymers of synthetic origin.^17,18^

The design of new biocompatible tissues has shown that interest in biopolymers has risen due to their benefits, including cytocompatibility and nontoxic degradation. Biopolymeric composites, whether organic or synthetic, possess predetermined physicochemical characteristics and porosity6.In this study, we propose the development and investigation of a novel hybrid polymeric matrix scaffold made of gelatin and hydroxyapatite loaded with doxycycline. The hybrid polymeric matrix system added with an active agent, such as doxycycline, is not fully understood. This combination facilitates a controlled degradation profile, drug release, and antimicrobial properties, and enhances the environment for cellular adhesion and proliferation within a single material, as inflammatory processes induced by infections can disrupt and delay these processes. We describe these matrices physically and chemically and test their antimicrobial activity against bacterial strains of (ATCC, 23235) bacterial strains. We also tested their osteogenic activity in vitro by increasing cell growth in preosteoblasts MC3T3 (ATCC, CRL-2593) and fibroblasts L929 (ATCC CCL-1).

## 2 Methods and Materials

### 2.1 Synthesis of the Composite Hydroxyapatite–Gelatin

The synthesis of hydroxyapatite and gelatin nanocomposites, containing doxycycline, was developed based on the protocols proposed by Kim et al.^16^ The first step is to solubilize 2,3006 g of monobasic ammonium phosphate [(NH_4_)·H_2_PO_4_] in 75 mL of deionized water [0.27 M] at 45 °C. Afterward, 3.75g (5% *w/v*) of type B gelatin powder was added to the solution under stirring for 1 h and a half at a temperature of 45 °C. The pH of the solution was then adjusted to 10 using ammonium hydroxide (NH_4_OH) 25% v/v. The second solution was prepared using 7,8556g of calcium nitrate tetrahydrate [Ca­(NO_3_)_2_]·4H_2_O in 60 mL of deionized water [0.55 M] at 45 °C. Afterward, 3,0g (5% w/v) of type B gelatin powder was added to the solution under stirring for 1 h and a half at a temperature of 45 °C. The Ca and P precursors were dissolved in distilled water separately, at the mole fraction ratio [Ca]/[P] = 1.67. Then, carefully by the drip method, 1 drop every 2 s, the first solution (ammonium phosphate) was poured over the second solution (calcium nitrate). This process was done by maintaining vigorous stirring and temperature at 45 °C for 24 h. At the end of the previous stage, the final solution was frozen in the refrigerator at −20 °C for storage.

### 2.2 Preparation of the Porous Composites to Study the Optimal Amount of Cross-Linker

The HA–gel solution stored was refrigerated under the temperature of −20 °C for 24 h and then lyophilized for 72 h. The ratio of composite to solvent was set at 1% (w/v) and 5% (w/v) based on preliminary experimental tests that aimed to maintain the pore structure and compare the physicochemical and drug release from the materials. The amount of drug was set at 0.7% (w/v), and the amount of cross-linking agent varied from 0.1% (w/v) to 1.0% (w/v) to the solvent, for which a solvent based on acetone and water (4:1 vol) was used at a temperature of 4 °C for 24 h. At the end of the cross-linking time, the samples were completely submerged in 96% ethanol for 1 h, followed by 70% ethanol for 30 min, and rinsed with distilled water three times to remove chemical residues and neutralize the pH. At the end of the wash, the samples were again frozen at −20 °C for 24 h and lyophilized for 48 h obtaining cross-linked porous composites for the study of the optimal amount of cross-linking agent.

### 2.3 Preparation of the Nanocomposite Porous Composites and Drug Loading

The stored HA–gel solution was taken for 24 h at a temperature below −20 °C and was lyophilized for 72 h. The composite-to-solvent ratio was set at 1% (w/v) and 5% (w/v) from preliminary tests to maintain the structures of the pores and compare the physicochemical and drug release from the materials. The amount of cross-linking agent was set at 0.7% (w/v), and the amount of drug varied from 0.3% (w/v) to 1.2% (w/v) to the solvent, for which a solvent based on acetone and water (4:1 vol) was used at a temperature of 4 °C for 24 h. At the end of the cross-linking time, the samples were completely submerged in 96% ethanol for 1 h, followed by 70% ethanol for 30 min, and rinsed with distilled water three times to remove chemical residues and neutralize the pH of the composites. At the end of the wash, the samples were again frozen at a temperature below −20 °C for 24 h and lyophilized for 48 h obtaining cross-linked porous composites for the characterization tests and *in vitro* tests. The identification of the composites is summarized in Table 1.

**Table 1:** Composition of the Composites

identification	composition
gelatin hydrogel – HA	HA and noncross-linked gelatin
composite 1	HA, gelatin and EDC (1% (*w*/*v*))
composite 1.1	composite 1 doxycycline (0.3% (*w*/*v*))
composite 1.2	composite 1 and doxycycline (0.7% (*w*/*v*))
composite 1.3	composite 1 and doxycycline (1.2% (*w*/*v*))
composite 2	HA, gelatin and EDC (5% (*w*/*v*))
composite 2.1	composite 2 and doxycycline (0.3% (*w*/*v*))
composite 2.2	composite 2 and doxycycline (0.7% (*w*/*v*))
composite 2.3	composite 2 and doxycycline (1.2% (*w*/*v*))

The composites containing the drug were not physicochemically characterized, due to the low concentration of the drug, which generates a biological difference, but not a physicochemical in the material. For *in vitro* tests, the composites were sterilized by several washes with 70% alcohol, followed by 30 min under UV light on each side.

### 2.4 Bacterial culture

Strains of () (ATCC 23235) were used for the test. It was grown in aerobiosis, in brain heart infusion broth (BHI), at 37 °C for 24 h. To reach the optical density (OD600) and 0.08 absorbance in a spectrophotometer, equivalent to 0.5 in the McFarland scale (about 1 × 10^8^ colony forming units (CFU)/mL).

### 2.5 Cell Culture

Mouse fibroblasts L929 (ATCC CCL-1), and murine preosteoblasts MC3T3 (ATCC, CRL-2593) were used for tests. They were grown in cell culture flasks containing Dulbecco’s modified Eagle’s medium (DMEM) high glucose (4500 mg·L^–1^), supplemented with 10% FBS (fetal bovine serum), 1% antibiotic/antimycotic (penicillin 10,000 units.mL^–1^/ streptomycin 10 mg.mL^–1^), 1% l-glutamine and 1% nonessential amino acids. All cells were incubated in a humidified incubator containing 5% CO_2_, and a temperature of 37.5 °C.

### 2.6 Applied Materials and Instruments

#### 2.6.1 Reagents

The following reagents were used:

Monobasic ammonium phosphate, molecular formula: (NH_4_)·H_2_PO_4_, analytical grade, ≥99.0% (RT), molar mass: 115.03 g/mol, manufacturer: Dinâmica, batch: 98043.

Calcium nitrate tetrahydrate, molecular formula: Ca­(NO_3_)_2_·4H_2_O, analytical grade, ≥99.0% (RT), molar mass: 236.15 g/mol, manufacturer: Dinâmica, batch: 118968.

Type B bovine gelatin, appearance: light yellow powder, manufacturer: Sigma-Aldrich, batch: G6650.

1-Ethyl-3-(3-dimethylaminopropyl)­carbodiimide hydrochloride, molecular formula: C_8_H_1__7_N_3_·HCl, analytical grade, ≥99.0% (RT), molar mass: 191.70 g/mol, appearance: off-white powder, manufacturer: Sigma-Aldrich, batch: E7750–5G.

Doxycycline, molecular formula: Ca­(NO_3_)_2_·4H_2_O, analytical grade, ≥99.0% (RT), molar mass: 444.4 g/mol, appearance: yellow powder, manufacturer: Araújo Compounding Pharmacy; on demand.

Alkaline phosphatase: LabTest ref 79.

Other reagents include ammonium hydroxide, acetone, distilled water, Milli-Q water, and PBS.

#### 2.6.2 Instruments

Fourier transform infrared spectroscopy (FT-IR) spectra were obtained using a PerkinElmer FT-IR Spectrometer Frontier located at the Mass Spectrometry Laboratory, Department of Chemistry, UFMG. Samples were analyzed in the range of 4000–500 cm^–^^1^.

Thermogravimetric analysis (TGA/DTG) curves were obtained using a TA Instruments SDT Q600 analyzer at the Molecular Encapsulation and Biomaterials Laboratory (LEMB/DQ-UFMG). Samples were placed in alumina crucibles with a mass between 5 and 10 mg. Equipment operation parameters are as follows: nitrogen flow rate of 100 mL·min^–^^1^ and a heating rate of 10 °C·min^–^^1^ up to 600 °C.

X-ray diffraction (XRD) patterns were acquired using a Rigaku D/MAX Ultima automatic diffractometer at the X-ray Diffraction Laboratory, Nuclear Technology Development Center, equipped with a θ–θ goniometer and a copper X-ray tube (Kα radiation), in the 2θ range of 10 to 70°, with a scan rate of 2°·min^–^^1^.

The materials were freeze-dried in a Savant Modulyo D-freeze-dryer, Thermo Electron Corp, located in the Inorganic Chemistry Laboratory of the Department of Chemistry at UFMG.

Scanning electron microscopy (SEM) images were obtained using the FEI Quanta 200 FEG scanning electron microscope at the Microscopy Center, UFMG.

### 2.7 Statistical analysis

The tests were conducted in triplicate in order to ensure the reliability of the results. The software OriginPro – Version 2018 was used to perform the statistical analysis. Initially, the data were subjected to tests for normality and homoscedasticity assumptions. When these assumptions were met, one-way analysis of variance (ANOVA) was applied, followed by Fisher’s least significant difference (LSD) test for post hoc comparisons when the *p*-value was <0.05.

For data that did not meet the assumptions, the Kruskal–Wallis test was used, followed by the Dwass–Steel–Critchlow–Fligner (DSCF) multiple comparisons test. In all analyses, statistically significant differences were considered at *p* < 0.05 (*), *p* < 0.01 (**), and *p* < 0.001 (***).

## 3 Results and Discussion

### 3.1 Physicochemical Characterization of the Composites

#### 3.1.1 Composite Morphology

The morphology of the non-cross-linked HA-gel hydrogel and the composites was evaluated by scanning electron microscopy (SEM) using an acceleration tension of 15 kV and magnifications of up to 5000×. The micrographs presented are in Figure 1.

**Figure 1:**
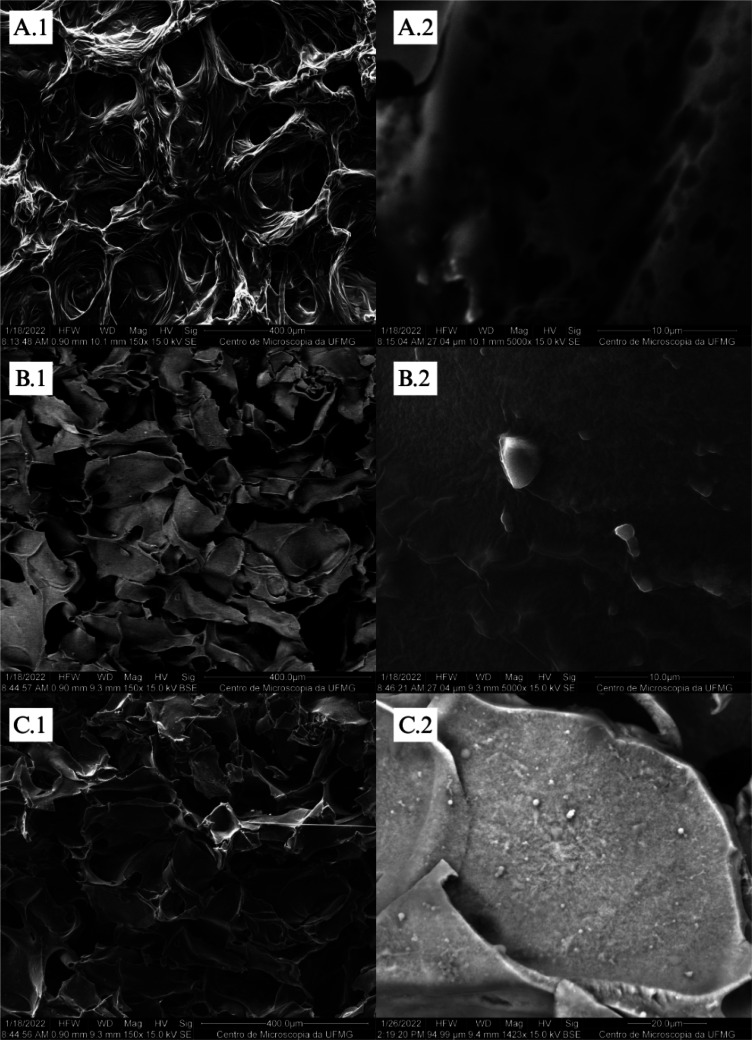
SEM micrographs of HA-gel without cross-linking at 400 (A.1) and 10 μm (A.2) composite 1. Cross-linking at 400 (B.1) and 10 μm (B.2) composite 2. Reticulated at 400 (C.1) and 10 μm (C.2).

The non-cross-linked HA-gel hydrogel has a homogeneous, well-developed pore structure with an oval configuration. Then, in sections B.1 and C.1 (Figure 1), it is possible to find out the changes in the structure after cross-linking the material. The material structure becomes irregular with a new laminated configuration, and the pore size decreases. As the pore size of hydrogel matrices decreases with the levels of cross-linking, such a decrease can be attributed to the used cross-linking agent (EDC), which participates in the reaction between molecules containing free carboxylic and amine groups to form amide bonds.^19^

The ionic precursors of Ca and P readily hybridize to gelatin chains by binding to gelatin functional groups in HA synthesis. Subsequent mixing of both solutions under the adjusted conditions facilitated the formation of HA nanocrystals. The HA nanoparticles were distributed homogeneously throughout the gelatin network, and the distribution was not affected by gravity, presumably due to the gelatin viscosity and the rapid freezing of the composite at the end of the synthesis.

#### 3.1.2 X-ray Diffraction (XRD)

X-ray diffraction (XRD) was performed to determine the degree of crystallization of the hydroxyapatite crystals synthesized within the gelatin network, using a copper tube and CuKα radiation = 1.54051 Å, operating at 30 kV and 30 mA, with variation of the 2θ angle from 10 to 70° and a scan rate of 2θ.min^–1^.

Analyzing the diffractograms is possible to verify the characteristic diffraction peaks of hydroxyapatite at 25.68, 28.85, 31.78, 32.12, 32.81, 33.85, 39.77, 46.71, and 49.40°.

The XRD diffractograms shown in Figure 2A indicate the presence of characteristic HA peaks in the nanocomposites, which show the formation of the compound, although some of the peaks were not distinct due to the masking effect of the gelatin matrix at low levels of HA particles. The weak division of the HA peaks between 31.78 and 32.12° corresponding to the (211) and (112) planes can be attributed to a low crystallinity, as also supposed by other works.^20^

**Figure 2:**
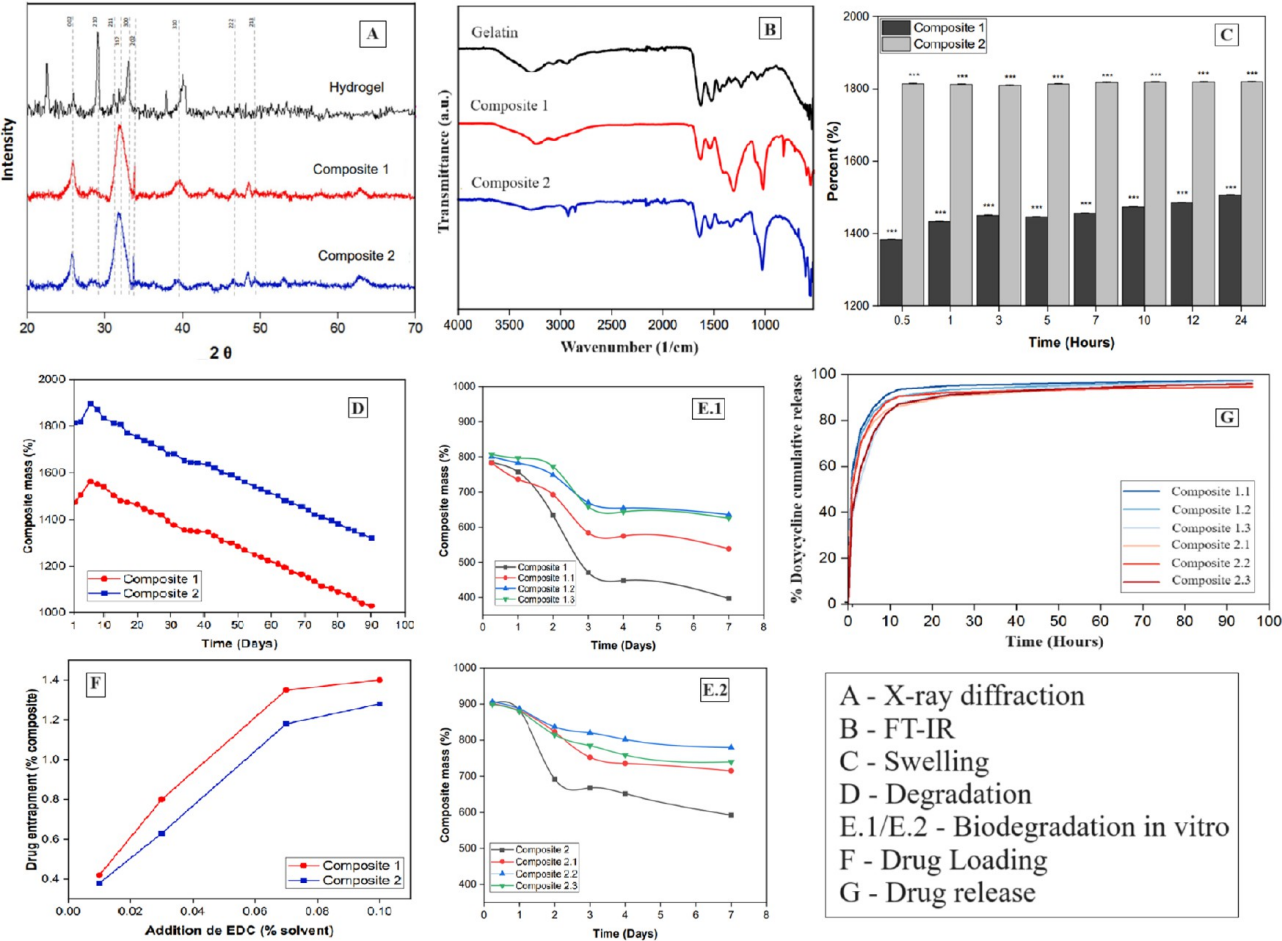
(A) X-ray diffractograms of the hydrogel and cross-linked composites. (B) Absorption spectra in the infrared region of the gelatin and its HA composites. (C) Swelling percentage of composites containing cross-linked HA and gelatin ANOVA Statistically significant difference *P* < 0.001 at each experimental time. (D) Degradation profile of composites containing cross-linked HA and gelatin, in physiological solution (PBS, pH 7.4); comparing both groups, there was a statistically significant difference *P* < 0.001 at each experiment time. (E) Degradation profile of composites containing cross-linked HA and gelatin, in enzymatic solution with collagenase type I (PBS, pH 7.4). (F) Drug loading and release profile using different EDC concentrations, in physiological solution (PBS, pH 7.4). (G) Drug release profile present in composites containing cross-linked HA and gelatin, in physiological solution (PBS, pH 7.4).

#### 3.1.3 Fourier-Transform Infrared Spectroscopy (FT-IR)

Fourier-transform infrared spectroscopy (FT-IR) was performed to investigate the main functional groups present in gelatin and its composites with HA. The spectra were obtained from KBr pellets in the region of 4000–550 cm^–1^, and the intensity of the peaks in the spectrum indicates the functional groups present in the material.

The FT-IR spectroscopy in Figure 2B highlights the main functional groups that make up each material. Pure gelatin showed OH bands (∼3450 cm^–1^), amide I at ∼1650 cm^–1^ (C=O bond stretching), amide II at ∼1550 cm^–1^ (N–H bond stretching and C–H bond stretching), and amide III at ∼1250 cm^–1^ (C–N bond tuning in phase with N–H bond bending). Furthermore, the bands present at ∼1380 and ∼1440 cm^–1^ are also due to amino acids in the gelatin structure, such as glycine, proline, and hydroxyproline.^21–23^

In the HA-Gel nanocomposites, the PO_4_ bands (∼570, ∼600, ∼960, and ∼1030–1090 cm^–1^), and OH (∼630 cm^–1^) were seen together with the gelatin bands, and some low-intensity bands possibly appeared due to the drying of the material. Based on FT-IR results, it is possible to state that gelatin, even when denatured from collagen, retains considerable characteristic biological groups, mainly amino acids. Furthermore, the amide bands suggest that gelatin maintains a high level of α-helix structure.^24^ This structural evolution was facilitated in part by gelatin cross-linking, during which the gelatin structure was ordered and the triple helix structure recovered to a high degree.^25^ Therefore, cross-linking of the gelatin is very important, and without it, the gelatin network will collapse within a fluid due to hydrolytic and thermal degradation and its loose structure.

#### 3.1.4 Total Porosity

The porosity of the composites was determined through the real and apparent densities. The determination of the real density is carried out with a He pycnometer, whose atomic radius is so small that it can penetrate the open porosity of the composites.

The apparent density was determined from a volumetric pycnometer of 10 mL and the known mass of the composites. Initially, the empty pycnometer was weighed; then the pycnometer was filled with mercury and finally filled with the sample and mercury. The apparent density was determined using eq 1

$$\rho_{\mathrm{apparent}}=\frac{m_{1}}{(m_{1}+m_{2})-m_{3}}$$ 1

*m*_1_ = Known mass of the composite

*m*_2_ = Pycnometer mass filled with mercury

*m*_3_ = Pycnometer mass filled with mercury and the composite *m*_1_

With both densities calculated, the porosity was determined by finding the densities of the composites using eq 2:

$$\mathrm{PT}\;(\%)=(1-\frac{\rho_{\mathrm{apparent}}}{\rho_{\mathrm{real}}})\times 100$$ 2

The total porosity of the composites is shown in Table 2. Composite 1 has a porosity of 72.97% and composite 2 has a porosity of 81.92%, the results are as expected. According to the composite 1 variation (1% *w/v*), the effect of the cross-linker would be expected to be greater wherefore fewer pores along with smaller ones would be expected. On the other hand, composite 2, being 5% *w/v*, would not have an effect of the cross-linker on the material as strong, so larger pores would be expected, which is interesting, as adjusting the pore size and porosity would help to obtain similar density, strength and mechanical compatibility with bone tissues, which can effectively prevent osteonecrosis and osteogenesis deformity around the graft.^26^

**Table 2:** Densities and Porosities of the Composites

identification	ρ_real_	ρ_apparent_	porosity (%)
composite 1	0.026 ± 1.82 × 10^–3^	0.007028 ± 5 × 10^–6^	72.97
composite 2	0.039 ± 2.73 × 10^–3^	0.007053 ± 5 × 10^–6^	81.92

In general, both materials present an internal perforated porous structure, which in biomaterials is beneficial for the adhesion, compliance, and differentiation of mesenchymal stem cells. The porous structure also provides a high interfacial fixation area for vascularization and bone growth, promoting biological fixation of graft and bone.^27^

Although several studies in the literature do not clearly show the ideal porosity and pore size of bone implants for bone growth, a study by Chen^28^ showed that the ideal porous structure in terms of porosity and pore size of an SLM Ti6Al4 V ELI composite for biomedical implants was explored. Various composites with pore sizes of 500, 600, and 700 μm and porosities of 60 and 70% were prepared, and in vitro and in vivo experiments were carried out to evaluate the biological performance of the porous composites. The material that presented the best results was that with 60% porosity. In the present work, the material with the highest cell adhesion obtained is composite 1, which has a porosity of 72.97%, which suggests that gelatin-HA composites must have a porosity of less than 80%, and possibly, the optimal porosity could be lower than 70%.

#### 3.1.5 Swelling Test

To study the swelling capacity of the composites, the swelling test was carried out according to the methodology found in the literature.^7,29^ Samples from each scaffold group were weighed in triplicate and submerged in PBS (pH 7.5) at 37 °C for the swelling test. Measurements were taken at the experimental time points of 30 min, 1 h, 3 h, 5 h, 7 h, 10 h, 24 h, 48 h, and 72 h. At each time point, the scaffolds were removed from the PBS, placed on filter paper for 1 s to remove excess surface liquid, and then weighed. The swelling ratio was calculated using eq 3 and was expressed as mean ± SD (*n* = 3) (standard deviation).

$$\%\mathrm{swelling}=\left[\frac{\left(W_{t}-W_{i}\right)}{W_{i}}\right]\times 100$$ 3

*W*_*i*_ = Mass of each lyophilized composite before submerging it into the PBS solution.

*W*_*t*_ = Mass of each composite after the removal of PBS.

The swelling capacities of the composites are shown in Figure 2C.

Swelling behavior is one of the most important parameters to evaluate biomedical applicability. A higher rate of swelling typically results in a greater surface area for the diffusion of bioactive compounds and migration of cells into the composite 3D structure.^30,31^ The swelling capacity of the composites is shown in Figure 2C. Composite 1 was the one with the lowest swelling rate at all experiment times. Each of the freeze-dried composites was weighed 20 mg before being immersed in PBS. Between times of 0.5 and 24 h, composite 1 showed a 123% increase in swelling, while composite 2, at the same time variation, showed an increase of only 5%. This increase is modest; in addition, in the case of composite 1, during the first 30 min in contact with the PBS, it reached 90% of its swelling limit and composite 2 reached practically 100%. Initially, a linear increase between the proportion of swelling and the immersion time until reaching equilibrium would be expected, so it is inferred that equilibrium was reached during the first 30 min of immersion of the composites in PBS.

Furthermore, at the end of 24 h, composites 1 and 2 showed swelling rates of 1817.16 and 1506.11%, respectively, with a difference between them of 313.05%. The degree of cross-linking of each composite had a clear effect on the swelling balance of the composites. Finding that the swelling ratio of HA and gelatin composites decreased with an increasing degree of cross-linking. This result is in line with those in the literature.^32–34^

#### 3.1.6 Composite Degradation

The degradation of the gelatin-based composites was studied according to the methodology adapted from NINAN.^35^

Three samples from each composite group were weighed, separated, and placed in PBS (pH 7.5) at 37 °C. At experiment times 1, 3, 5, 7, 10, and 14 days. The composites were removed from PBS, washed in deionized water, and lyophilized. The dry mass of the composite was recorded. The percentage of degradation was calculated using eq 4 and was expressed as mean ± SD (*n* = 3).

$$\%\mathrm{de}\mathrm{grad}\mathrm{ation}=\left[\frac{\left(W_{0}-W_{1}\right)}{W_{1}}\right]\times 100\%$$ 4

*W*_0_ = Mass of each lyophilized composite before submerging into the PBS.

*W*_1_ = Mass of each composite after the removal of PBS.

The degradation study monitors the loss of mass as a function of the incubation period. The degradation curves are represented in Figure 2D, where 100% of the *Y* axis refers to the amount of composite weighed dry at the beginning of the test (20 mg).

The degradation curves are represented in Figure 2D. It is necessary to ensure that the degradation of the nanocomposite is not too fast as this does not favor the integrity of the composite before the regeneration of the new tissue. The group with EDC cross-linking agent at 0.7% *w*/*v* was optimal for modifying the gelatin structure so that its cross-linking was significant enough to improve drug release with a sufficiently slow degradation to the support structure necessary in bone regeneration. It was observed that the well-cross-linked composites remained for several months.

The HA-gel hydrogel without the cross-linking process was completely dissolved in PBS solution within 1 h. In contrast, when the material was cross-linked with EDC, in the test carried out for 90 days, the composites maintained their structures in the PBS solution with a mass loss of 30 and 34% of composite 1 and 2 respectively, from the maximum absorption of water reached on day 3 of the test, it is possible to verify that the cross-linking carried out provides the system with greater stability.

#### 3.1.7 Biodegradation *In Vitro*

The enzymatic degradability of the composites was evaluated using the type I collagenase enzyme with a study by monitoring mass loss during the incubation period. The tests were carried out according to the methodology adapted from Ng et al.^36^

Samples of approximately 5 mg of the lyophilized and sterilized composites were incubated at 37 °C under agitation (60 rpm) in 2 mL of an enzymatic solution. The enzymatic solution was prepared by dissolving type I collagenase (200 U/mg, 17100–017, GIBCOTM) in 50 mM tris­(hydroxymethyl)­aminomethane hydrochloride (Tris/HCl) buffer containing 5 mM CaCl_2_ (pH 7.4) up to a concentration of 30 U/mL.

The samples were incubated for 6 h and 1, 2, 3, 4, and 7 days. After each period, they were removed from the solutions, placed on filter paper for 1 s to remove the surface water, and weighed. The rate of mass loss was defined by eq 3. The degradation curves are represented in Figure 2E1,E2, where 100% of the *Y* axis refers to the amount of composite weighed dry at the beginning of the test (5 mg).

The mass loss of the composites observed in Figure 2E1,E2 is a function of the time they are submerged in the enzymatic solution, type I collagenase (30U/mL) in 50 mM Tris/HCl buffer containing 5 mM CaCl_2_ (pH 7.4).

Table 3 shows the percentage of degradation after 7 days from the start of the test, where it is possible to observe the difference between the composites with and without doxycycline.

**Table 3:** Composite Degradation after 7 Days

identification	degradation (%)	identification	degradation (%)
composite 1	49.32	composite 2	34.87
composite 1.1	29.21	composite 2.1	20.71
composite 1.2	20.53	composite 2.2	13.96
composite 1.3	22.50	composite 2.3	17.73

When analyzing the data, it is possible to observe a greater degradation in materials that do not contain doxycycline. Seven days after the start of the test, the mass loss of composites 1 and 2 was 49 and 35%, respectively. However, the composites that presented different concentrations of doxycycline had a biodegradation that varied from 14 to 29%, as doxycycline inhibits the collagenolytic activity of matrix metalloproteinases (MMP), which degrade the extracellular collagen matrix,^20^ which protects the matrix containing doxycycline from rapid degradation. The degradation process of composites 1 and 2 is due to the breakdown of amide bonds in the collagen peptide caused by the collagenase enzyme.^37^

Studies have shown that doxycycline has the potential to improve the control of other inflammatory processes, such as skin diseases and chronic wounds.^38^

#### 3.1.8 Cross-Linking

The amount of cross-linking agent was carried out to determine the ideal % (*w*/*v*) of cross-linking agent needed in the composites, ensuring the ideal absorption of the drug, without the need to use too much cross-linking agent. For this test, cross-linked composites with different concentrations of cross-linking agent and a fixed concentration of the drug were used.

In triplicate, 20 mg of composites were placed in 2 mL of PBS. Then, the solutions were kept under agitation in an incubator at 37.5 °C. At the periods of 0, 1.5, 3, 5, 7, 9, 12, 24, 36, 48, 72, and 96 h, the 2 mL of liquid was removed and replaced by new PBS. The aliquots were analyzed in an Ultraviolet–visible (UV–vis) spectrophotometer at 270 nm for doxycycline, using a white PBS control. For the determination of doxycycline concentrations, a calibration curve was made with known concentrations using PBS as white. Measures were taken with a UV–vis spectrophotometer at a wavelength of 270 nm. Cross-linked HA-gel nanocomposite composites can potentially deliver bioactive molecules and can yield therapeutic effects. As a first step toward the application of drug release from composites, doxycycline was loaded and cross-linked, and the drug loading profile was investigated as shown in Figure 2F.

Both drug capture and release profiles were greatly affected by the degree of cross-linking (Figure 2F). During cross-linking, doxycycline dissolved in solvent migrated and was absorbed into the HA-gel network due to the difference in concentration and was trapped within the structure.

The carbodiimide derivative (EDC), chosen as the cross-linking agent in this article, is much safer in terms of toxicity than the glutaraldehyde conventionally used in gelatin cross-linking, as it does not leave byproducts during cross-linking.^39^ When the HA-gel networks were poorly cross-linked, the composites could not capture the drug efficiently; Instead of capturing it, the material released it into the cross-linking solution due to its loose structure. The carbodiimide derivative (EDC), as a cross-linking agent, acted to unite the carboxyl and amine groups in the gelatin amino acids and formed longer bonds to form amide chains.^39^ As a result, the gelatin network became stronger chemically and thermally as well as more structurally compact. With an increase in EDC up to 0.7% *w*/*v*, the composite had stability in the amount of drug capture and release in both composites, but with greater additions of cross-linker, insignificant changes were observed, which is why it is not feasible to cross-link the materials at a rate greater than 0.7% *w*/*v.*

The difference between composites 1 and 2 is the mass quantity of hydrogel added concerning the volume of solvent at the time of cross-linking, being 1% *w*/*v* and 5% *w*/*v*, respectively. It is observed that composite 1 presents a greater amount of drug than composite 2. This is because the percentage of cross-linker and the percentage of drug depend on the percentage of solvent, for which composite 1 has contact with a greater amount of EDC and drug than composite 2.

#### 3.1.9 Doxycycline Release

The controlled release of doxycycline in the composites was carried out to determine the kinetics of drug release. In triplicate, 20 mg of composites were placed in 2 mL of PBS. Then, the solutions were kept under agitation in an incubator at 37.5 °C. At the periods of 0, 1.5, 3, 5, 7, 9, 12, 24, 36, 48, and 72 h, the 2 mL of liquid was removed and replaced by new PBS.

To determine the doxycycline concentrations, a calibration curve was made with known concentrations using PBS as white. All of the measurements were taken with a UV–vis spectrophotometer at a wavelength of 270 nm. The drug release profile from the composites is represented in Figure 2G, as well as the amounts of drug released in Table 4.

**Table 4:** Concentration of Doxycycline Released from Each Composite

identification	concentration (mg/L)	identification	concentration (mg/L)
composite 1.1	97.42	composite 2.1	81.52
composite 1.2	135.70	composite 2.2	132.44
composite 1.3	193.91	composite 2.3	172.80

The system shows an explosive release profile with approximately 60% of the total drug content released in the first 3 h of release and stabilizing after 12 h (Figure 2G). The drug release profile is mainly affected by the fluid absorption and degradation of the gelatin present in the composite. In the first few hours, the drug is released by diffusion: when the composite absorbs fluids and swells, the drugs diffuse more easily. Second, the drug is released directly by the loss of material and, in these composites, the loss of material occurs both through the degradation of gelatin and the ionic release of HA.^40^

Drug release from composites appears to follow the loss of material. However, although the material loss was practically negligible in the first 100 h of the degradation test, drug release showed a reduced rate over time, confirming that drug release did not result directly from material loss, but rather depended on much of the diffusion mechanism. Compared to the degraded number of composites, the drug release was much greater; when cross-linked with 0.7% EDC, the mass loss was less than 1%; however, the amount of doxycycline released was 85 to 95%.

Biodegradable polymers, such as polylactic acid, gelatin, or chitosan, are used as matrices for ceramic particles or as adjuvants for calcium phosphate cement. The use of these polymers can introduce tailored biodegradation/drug release to the ceramic material.^41^ The HA-gelatin nanocomposite composites exhibited a well-controlled drug release profile, showing a cargo-dependent drug release. When the initial addition of doxycycline was normalized, the release profile was almost identical. This is of particular importance in drug release as the exact release can be controlled simply by changing the amount of the drug charge.

### 3.2 Biologic Tests *In Vitr* *o*

#### 3.2.1 Antibacterial Analysis

The antibacterial properties of the composites were investigated by minimum inhibitory concentration (MIC) and minimum bactericidal concentration (MBC) methods, where sample groups were weighed at 10 mg, submerged in 1 mL of BHI broth, and incubated for 24 h at 37 °C to produce an eluate for each group. After the incubation period, a dilution was made initially 50 times, and in this initial dilution, a dilution of 2:1 was made 6 times. 50 μL of each eluate was added in triplicate to the plate, along with 50 μL of bacteria, and incubated for 24 h, based on Orooji protocol with modifications.^42^ After the incubation period, measurements were taken in a UV–vis spectrophotometer at a wavelength of 600 nm to help confirm the MIC.

Bacterial growth was represented in percentage, with 100% of the reading obtained from bacteria with free growth and 0% or control with chlorhexidine in the UV–vis spectrophotometer at a wavelength of 600 nm. To evaluate the minimum inhibitory concentration range of the composites, the bacterial growth inhibition test was performed on subcultures of bacterial strains of (). Table 5 shows the results of the MIC and MBC concentrations of each one of the composites.

**Table 5:** Inhibitory Concentration Range of Each Composite Proportional to Released Doxycycline

identification	MIC (mg/L)	MBC (mg/L)	identification	MIC (mg/L)	MBC (mg/L)
composite 1.1	0.24	≥0.24	composite 2.1	0.38	≥0.38
composite 1.2	0.33	≥0.33	composite 2.2	0.33	≥0.33
composite 1.3	0.23	≥0.23	composite 2.3	0.41	≥0.41

Antimicrobial activity was evaluated using as it is the main cause of bacterial infection in humans, causing everything from soft tissue infections to pneumonia, sepsis, and septic shock syndrome.^43^ Penetration into deeper tissues is produced by invasive procedures in healthcare environments, such as the introduction of prostheses, generating chronic infectious processes such as osteomyelitis,^44–46^ considering the information above, this bacterium was chosen to perform the bacterial inhibition test.

Analyzing Table 5, it is possible to notice that the minimum inhibition concentration of the doxycycline within the composites is 0.48 mg/L for composite 1.1, 0.33 mg/L for composite 1.2, 0.23 mg/L for composite 1.3, 0.38 mg/L for composite 2.1, 0.33 mg/L for composite 2.2, and 0.41 mg/L for composite 2.3, and the minimum bactericidal concentration is within the range of 0.48–0.24 mg/L for composite 1.1, 0.33–0.65 mg/L for composite 1.2, 0.23–0.45 mg/L for composite 1.3, 0.38–0.8 mg/L for composite 2.1, 0.33–0.65 mg/L for composite 2.2, and 0.41–0.82 mg/L for composite 2.3.

According to Figure 2, at least 1 ppm is released at all times analyzed (24–48, 48–72, and 72–96 h), which guarantees that the minimum inhibitory concentration (MIC) is maintained over time. The minimum inhibitory concentration that was found is superior to what was reported in other studies,^47^ where the MIC of doxycycline for S. aureus is 1 ppm, that is, the released doxycycline will be sufficient to maintain bacterial inhibition over time.

#### 3.2.2 Composites Cytotoxicity

All of the sample groups were weighed at 10 mg, submerged in 1 mL of nonsupplemented cellular culture medium, and incubated for 24, 48, and 72 h at 37 °C and in an ambiance with a controlled level of 5% of CO_2_ to produce an eluate for each group. After each period a dilution of every eluate was made at a ratio (1:1) using 300 μL of the initial eluate and 300 μL of the medium, thus obtaining a second eluate of 5 mg/mL 100 μL of each eluate were added in quintuplicate to the plate and incubated for 24 h. After the incubation period, the plates were washed twice with sterile PBS solution, and the medium was replaced with DMEM low glucose without phenol red, supplemented with 10 μL of 3-[4,5-dimethylthiazol-2-yl]-2,5-diphenyltetrazolium bromide (MTT) reagent at a concentration of 5 mg/mL, dissolved in PBS. The plates were protected from light due to the photosensitivity of MTT. After 4 h of incubation at 37 °C, 5% CO_2_, to form blue crystals, 50 μL of sodium dodecyl sulfate (SDS) detergent solution was added to stop the formation of the crystals, and after 15 min of shaking, readings were taken on a UV–vis at a wavelength of 570 nm. Cytotoxicity was calculated using eq 5), with the control group corresponding to untreated cells.

The cytotoxicity assay was performed using an MTT colorimetric test, which determines cell viability by measuring the activity of the mitochondrial reductase enzyme in living cells.

With a confluence of 80%, the cells were washed with PBS and subsequently trypsinized with 0.25% trypsin/ethylenediaminetetraacetic acid (EDTA) for 4 min and transferred to 96-well plates with a concentration of around 6 × 10^3^ cells/well, each plate was incubated for 24 h at 37 °C and 5% CO_2_.

For the test, 10 mg of the sample were weighed and submerged in 1 mL of nonsupplemented cell culture medium and incubated for 24, 48, and 72 h at 37 °C, and 5% CO_2_. After each period, a dilution of each eluate was made, with a ratio (1:1) using 300 μL of the initial eluate and 300 μL of the medium, thus obtaining a second eluate of 5 mg/mL. 100 μL of each eluate, was added, in quintuplicate on the plate, and incubated for 24 h. After the incubation period, the plates were washed twice with sterile PBS solution, and the medium was changed to DMEM low glucose without phenol red added with 10 μL of MTT reagent, with a concentration of 5 mg mL^–1^, dissolved in PBS. The plates were protected from light due to the photosensitivity of MTT. After 4 h of incubation at 37 °C, 5% CO_2_, for the formation of blue formazan crystals, 50 μL of sodium dodecyl sulfate (SDS) detergent solution was added. After completing 15 min of agitation, measures were taken on a UV–vis at a wavelength of 570 nm. Cytotoxicity was calculated using eq 5), the control group corresponds to the untreated cells.

$$\%\mathrm{cellular}\;\mathrm{viability}=[\frac{\mathrm{tested}\;\mathrm{absorbance}}{\mathrm{control}\;\mathrm{group}}]\times 100\%$$ 5

To find the percentage of viable cells, the control group was considered with 100% viability. Cellular cytotoxicity was classified according to the International Organization Standard (ISO 10993–5),^48^ described by Xiao,^49^ where grades 0 and 1 represent noncytotoxicity and grades 2, 3, 4, and 5 represent different levels of cytotoxicity, as shown in Table 6:

**Table 6:** Levels of Cytotoxicity

level	cellular viability
0	≥100%
1	≤99%
2	≤75%
3	≤49%
4	≤25%
5	= 0

The cytotoxicity test of the composites was tested indirectly in cultured L929 fibroblasts and MC3T3 preosteoblasts. For the test, an eluate at a concentration of 5 mg/mL was used. The cell viability results are represented in Figure 4A,B.

L929 fibroblast cells were treated with the media that had been in contact with the composites for 24, 48, and 72 h for 24 h. The cytotoxicity of the composites was evaluated by using the MTT colorimetric method.

MC3T3 preosteoblastic cells were treated with the media that were in contact with the composites for 24, 48, and 72 h for 24 h. The cytotoxicity of the composites was evaluated using the MTT colorimetric method.

Figure 3 shows that none of the composites presented toxicity to cell lines. The results obtained show more than 87% cytocompatibility of the composites with cells, presenting degree 1 or 0 of cytotoxicity according to the International Organization for Standardization (ISO) 10993–5,^48^ and the results are shown in Table 7.

**Figure 3:**
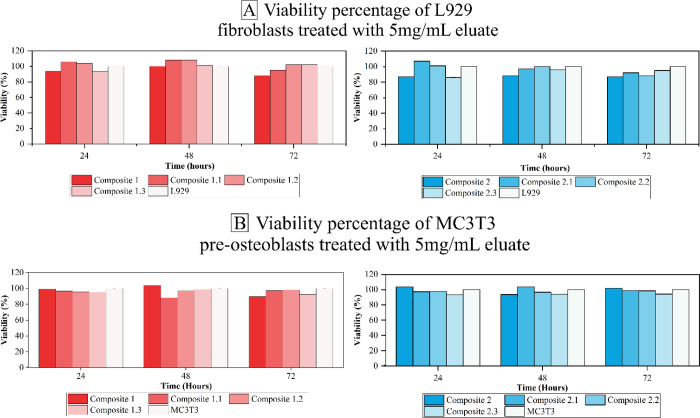
(A) Viability percentage of L929 fibroblasts treated with 5 mg/mL eluates containing composites HA-gelatin in different concentrations of doxycycline, at different experiment times, incubated for 24 h; (B) viability percentage of MC3T3 preosteoblasts treated with 5 mg/mL eluates containing composites HA-gelatin in different concentrations of doxycycline, at different experiment times, incubated for 24 h.

**Table 7:** Levels of cytotoxicity of the composites

levels of cytotoxicity				
		24 h	48 h	72 h
L929	1	1	1	1
	1.1	0	0	1
	1.2	0	0	0
	1.3	1	0	0
	2	1	1	1
	2.1	0	1	1
	2.2	1	0	1
	2.3	1	1	1
MC3T3	1	1	0	1
	1.1	1	1	1
	1.2	1	1	1
	1.3	1	1	1
	2	0	0	0
	2.1	1	1	1
	2.2	1	1	1
	2.3	1	1	1

The low cytotoxicity of composites based on fibroblasts is significant as these cells reach the site of injury in the early stages and proliferate quickly, leading to healing.^50^ L929 cells have already been tested on other composites and hydrogels containing the components used and components similar to those used in the composites, and the results were similar to those obtained in this experiment.^31,51,52^

In a study by Shanmuganathan et al.,^52^ chitosan microspheres loaded with doxycycline were developed, where the MTT test on fibroblasts showed that the microspheres loaded with doxycycline can improve the percentage of cell viability compared to the pure drug, by generating a controlled release of the drug.

The MTT test on MC3T3 preosteoblastic cells showed that none of the composites presented toxicity to the cell lines. The results obtained show more than 82% cytocompatibility of the composites with the cells, presenting degree 1 or 0 of cytotoxicity according to ISO 10993–5.^48^

As evidenced, none of the eluates showed cytotoxicity, which is why it was decided to carry out a complementary test. MC3T3 preosteoblastic cells were treated with the media that were in contact with the composites for 24, 48, and 72 h for 4 days in a higher concentration eluate, 10 mg/mL. The cytotoxicity of the composites was evaluated using the MTT colorimetric method.

The complementary MTT test shown in Figure 4 is evident that the composites without doxycycline 1 and 2, together with the composites 1.1 and 2.1, which released 48, 71, and 40.76 ppm of doxycycline, respectively, continue to present low cytotoxicity, with grade 1 according to ISO 10993–5. However, materials that contain a greater amount of drug (composites 1.2, 1.3, 2.2, and 2.3) present low cell viability in a range between 19.6 and 63.7% with degrees of cytotoxicity between 2 and 4, as shown in Table 8 below.

**Figure 4:**
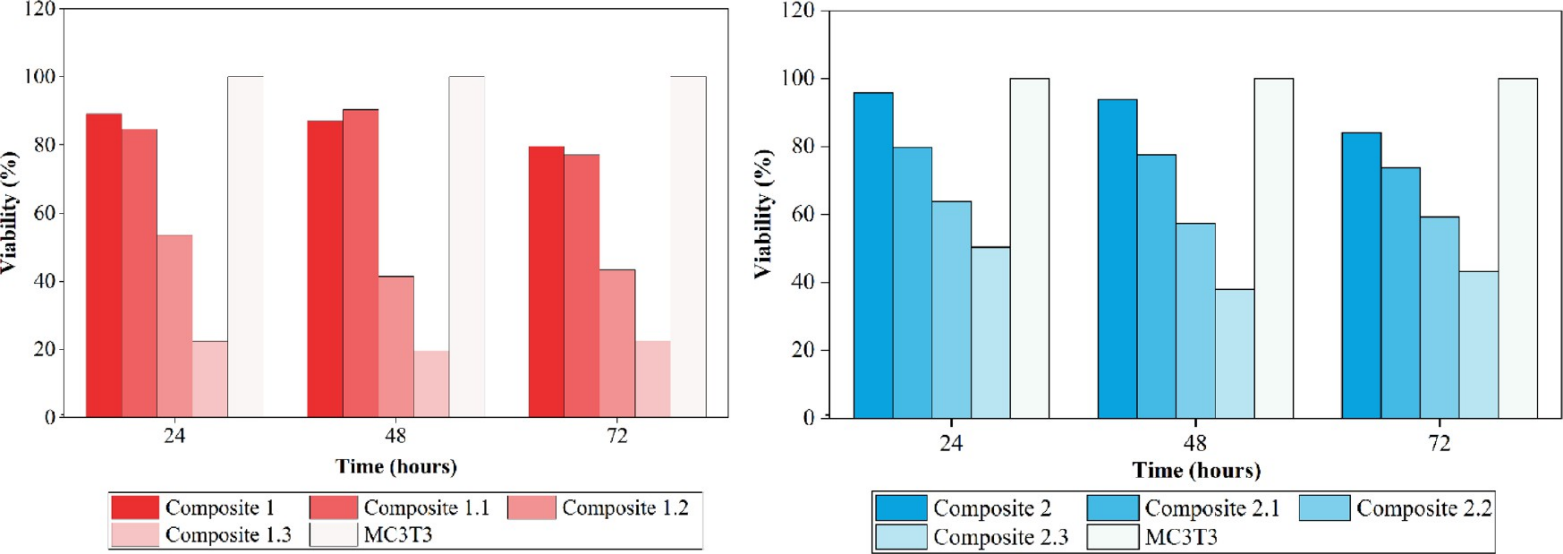
Viability percentage of MC3T3 preosteoblasts treated with 10 mg/mL eluates containing composites HA-gelatin in different concentrations of doxycycline, at different times, incubated for 4 days.

**Table 8:** Levels of cytotoxicity of the composites in contact with MC3T3 cells for 4 days

levels of cytotoxicity				
		24 h	48 h	72 h
MC3T3	1	1	1	1
	1.1	1	1	1
	1.2	2	3	3
	1.3	4	4	4
	2	1	1	1
	2.1	1	1	2
	2.2	2	2	2
	2.3	2	3	3

Hydroxyapatite and other calcium phosphate bioceramics are important for bone repair due to their excellent biocompatibility and bioactivity.^41^ MC3T3 cells have been previously tested on other composites and hydrogels containing doxycycline, pure or substituted HA, gelatin, and other components similar to those used in this study, and the results obtained in this experiment were corroborated by previous reports.^53–55^

The positive results were as expected as the materials used to manufacture the composites have already proven to be biocompatible. Furthermore, one of the main reasons why degrees 0 and 1 of cytotoxicity were obtained in both L929 fibroblasts and MC3T3 preosteoblasts is due to the controlled release of doxycycline. When doxycycline is used as a pure drug, there is a significant decrease in cell viability above 200 μg.^52^

The most cytotoxic material is composite 1.3, where the 10 mg/mL eluate showed a drug concentration of 193.91 mg/L (Table 4). Cytotoxicity is verified in the cellular micrographs represented in Figure 6, where cells in a state of apoptosis are visualized in composites 1.2, 1.3, 2.2, and 2.3, possibly due to the amount of doxycycline present in the composites. Studies show that high concentrations of doxycycline can decrease cell viability.^52^

#### 3.2.3 Cell Adhesion and Morphology

The cell adhesion test was performed using the methodology adapted from BEGAM et al.^56^ In this case, the test was performed with MC3T3 preosteoblastic cells. Initially, the samples from each of the composites were placed in triplicate in wells of a 24-well plate. In each well, 1 × 10^6^ cells were seeded drop by drop and the plate was incubated for 30 min at 37 °C, 5% CO_2_. After this time, 300 μL of the specific culture medium was added to each well and the plate was incubated again at 37 °C, 5% CO_2_, for 2 and 4 h. After each of the determined periods, cell adhesion was evaluated by collecting the culture medium from each well and counting the number of cells present in the supernatant. The number of adhered cells was quantified by subtracting the number of cells in the suspension from the initial concentration of seeded cells.

For the observation of cell morphology on the material, the hydrogel samples seeded with the MC3T3 cells were washed with sterile PBS twice, at an experimental time of 3 days. Then, the samples were fixed with 2.5% glutaraldehyde in 0.1 mol·L^–1^ phosphate buffer for 4 h. After removal of the fixative, 0.1 mol·L^–1^ of phosphate buffer was added. The samples in buffer were sent for secondary fixation, dehydration, critical point drying of CO_2_, and metallization of thickness 10 nm for subsequent analysis in SEM.

Cell adhesion was analyzed by two methods: cell counting and scanning electron microscopy. The result of the percentage of MC3T3 cells adhered to the material after 2 and 4 h is shown in Figure 5. The micrographs of the adhered cells are shown in Figure 6.

**Figure 5:**
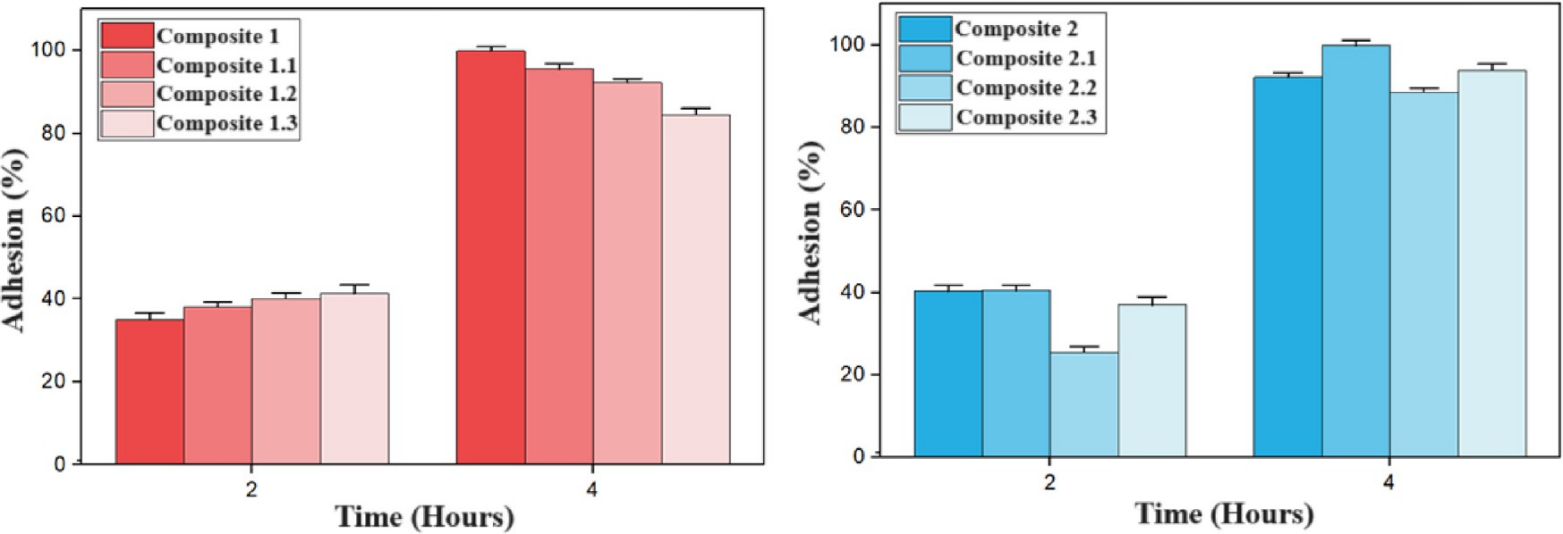
*In vitro* adhesion of MC3T3 preosteoblastic cells to composites. Kruskal–Wallis without statistically relevant differences.

**Figure 6:**
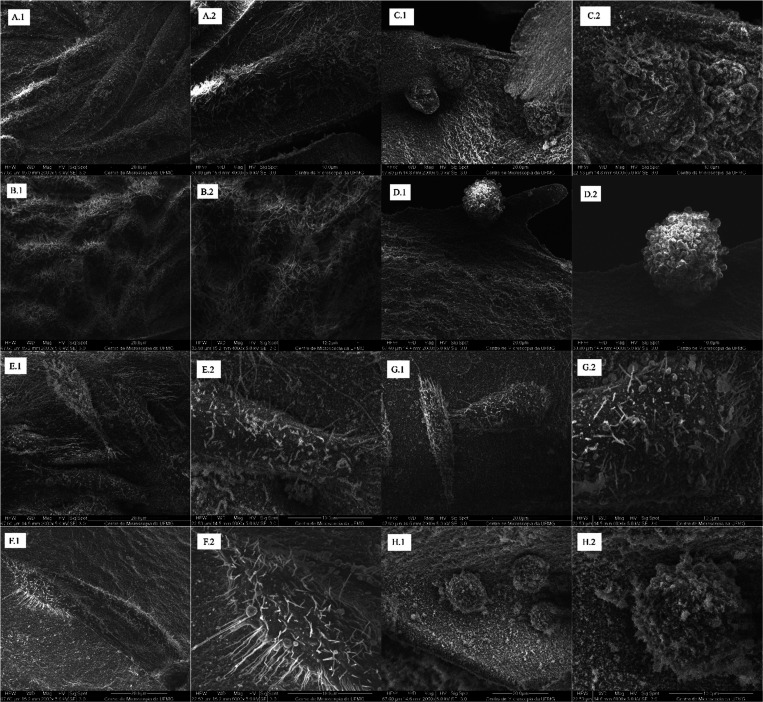
Adhesion of MC3T3 cells on composites 2.0 (E), 2.1 (F), 2.2 (G), and 2.3 (H). Magnifications at 2000× (_.1), 4000× (A.2, B.2, and D.2), and 6000× (C.2, E.2, F.2, G.2, and H.2).

After the experimental time of 2 h, the MC3T3 exposed on the composites showed approximately 40% adhesion, except for composite 2.2, which showed lower adhesion with 25.5%. At 4 h, there was adherence from 85 to 100%.

In the images obtained, the adhesion of MC3T3 cells can be observed after an experimental time of 3 days. Many branches can be seen in both cells. MC3T3 exhibited a spread morphology. The pores were completely filled by cells with cytoplasmic processes.

In the cell micrographs, many cytoplasmic extensions can be seen in both cells. MC3T3 cells exhibited a spread morphology and many processes. Doxycycline helped to obtain greater proliferation by comparing the B sections to the A sections in both variations of the material. B was the composite with the lowest concentration of doxycycline, and A was the composite used as a control that does not contain the drug.

One of the most important steps in the process of anchoring the interface of the material to bone is the adherence of osteoblasts to a material that has been created for bone application. Due to the fact that it fulfills its function of cell adhesion and because it assists with cell proliferation, the composites that were used as a control (Figure 6A,E) present cells that are elongated and differentiated. This indicates that the composite has the potential to regenerate tissue without the need for drugs. It was anticipated that this outcome would occur due to the fact that the HA particles are in nanometric size. This allows for a larger surface area of the cells to engage with the ceramic,^57^ which results in a higher level of contact and enhanced cell adhesion. There is a correlation between this result and the literature findings that were given by Yilmaz^58^ and Ma.^59^ Proliferation is also related to the effect of HA within polymeric matrices, Ca^2+^, Mg^2+^, and HPO_4_^2–^ ions are crucial for the metabolism of bone cells (osteoblasts and osteoclasts).^36^ Ca^2+^ activates the expression of bone-related proteins (such as osteopontin and bone sialoprotein) that are mediated by specific calcium-dependent channels and kinases. This mechanism increases osteoblast differentiation.^60^ Another possibility is the effect of gelatin on cell proliferation as it is a degradable component of collagen, being biocompatible, and its ability to bind and anchor cells.^61,62^ Both HA and collagen are the main components of the ECM in bones, used to regulate cellular morphology and function.^63^

#### 3.2.4 Alkaline Phosphatase (ALP)

Alkaline phosphatases (ALPs; EC 3.1.3.1) are dimeric, plasma-membrane-bound glycoproteins widely distributed across bacteria, animals, and plants. In vertebrates, they occur as tissue-specific isozymes, intestinal, placental, germ-cell, and a tissue-nonspecific form found in the liver, bone, and kidney, each encoded by a separate gene. As zinc-containing metalloenzymes, each monomer coordinates two Zn^2^^+^ ions and one Mg^2^^+^ ion in its active site, which not only drives the hydrolysis of a broad range of phosphate monoesters at alkaline pH but also stabilizes the enzyme’s fold and promotes subunit interaction. In higher organisms, the enzyme is tethered to the outer leaflet of the plasma membrane via a glycosylphosphatidylinositol (GPI) anchor at its C-terminus. Catalysis proceeds through a covalent phosphoserine intermediate: water attacks this intermediate under high-pH conditions to release inorganic phosphate, while in the presence of high concentrations of alcohols, a transphosphorylation reaction can occur, transferring phosphate to the organic acceptor.^64,65^ ALP is used as a biochemical marker to identify early osteogenic differentiation;^66^ hence, ALP activity was measured by the release of thymolphthalein resulting from the enzymatic hydrolysis of thymolphthalein monophosphate, following the protocol provided by the Labtest Diagnóstica ALP kit.

First, MC3T3 cells were seeded at a density of 1.5 × 10^4^ cells per well in 24-well plates containing the composites, and they were incubated at 37 °C with 5% CO_2_ for durations of 7, 14, and 21 days. At each time point (7, 14, and 21 days), the culture medium of the wells was discarded and double washed with sterile PBS; subsequently, they were filled with 2 mL of 0.2% Triton-X100 solution to promote cell lysis. After a 10 min incubation, the cell lysates were collected for ALP activity analysis.

The procedure began by mixing 800 μL of a solution containing HEDTA buffer (2.0 mmol/L, pH 10.4), zinc sulfate (1.2 mmol/L), and magnesium acetate (2.5 mmol/L) with 200 μL of a solution containing *p*-nitrophenylphosphate (60 mmol/L) and phenol (50 mmol/L). Next, 1 mL of this combined solution was mixed with 20 μL of cell lysate and incubated at 37 °C in a water bath for 10 min.

Absorbance was measured 2 times at 405 nm using a spectrophotometer: the first reading (A1) was taken after 1 min, followed by a second reading (A2) after 2 more minutes. The normalization of the data was made by the eq 6:

$$\frac{\Delta A}{\min}=\frac{\left(A2-A1\right)}{2}$$

ALP(U/L)=ΔA/min×2764 6

The results are given in Figure 7 below:

**Figure 7:**
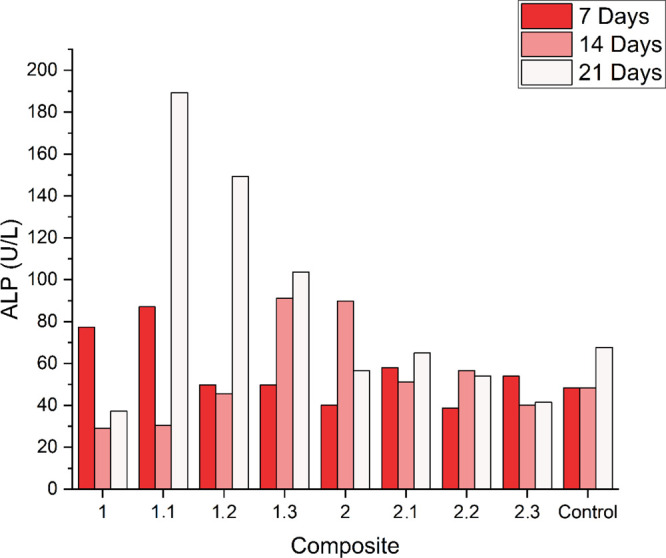
Alkaline phosphatase results expressed in U/L after normalization by eq 6. Kruskal–Wallis without statistically relevant differences.

The sample group with the highest expression of alkaline phosphatase was composite 1.1 at 21 days, indicating that low concentrations of doxycycline played a role in promoting the initial differentiation of preosteoblasts. These results are consistent with previous reports in the literature,^67,68^ which found that low concentrations of doxycycline were suitable for ALP expression.

The main limitations of this study lie in translating in vitro findings into an in vivo model. Factors such as determining the effective drug concentration in a controlled-release system present a paradox: increasing the concentration may enhance effectiveness against microorganisms, but it could also increase material toxicity.^69^ Furthermore, in vivo systems are far more complex than in vitro ones. The action of proteins and enzymes can accelerate both the release rate and the degradation of the material.^70^ Therefore, in vivo studies using these concentrations are necessary in order to optimize them.

Nonetheless, this article contributes by demonstrating that using doxycycline at a concentration of 0.3% (w/v), as in composite 1.1, can promote proliferation, adhesion, and early differentiation of preosteoblasts. This may offer a solution to the challenge of balancing toxicity and antimicrobial activity, given doxycycline’s collagenase-inhibiting properties, good biocompatibility, and retained antimicrobial activity.

## 4 Conclusions

Based on the data obtained, it is possible to conclude that a composite gelatin-HA with added doxycycline with a concentration of 0.3% (w/v) had great biocompatible properties and a biomimetic bone structure, ideal for tissue regeneration, such as porosity, degradation, and swelling, verifying that this composite designed together with the incorporation of doxycycline generate greater proliferation, adhesion, and early differentiation in preosteoblastic cells (MC3T3). Thus, the composite formed is an excellent alternative for the development of new composites for tissue engineering and drug loading. The physicochemical characteristics of the material favored a structure with drug release and degradation profiles compatible with the bone regeneration time. The addition of doxycycline to the composite inhibited the collagenolytic activity of MMPs, resulting in a composite with a degradation profile suitable for bone regeneration. The addition of doxycycline also gives material antimicrobial properties. The material surface of the composite positively favored preosteoblast proliferation, adhesion, and early differentiation.
